# Skin Autofluorescence Relates to Soluble Receptor for Advanced Glycation End-Products and Albuminuria in Diabetes Mellitus

**DOI:** 10.1155/2013/650694

**Published:** 2013-03-10

**Authors:** J. Škrha, J. Šoupal, G. Loni Ekali, M. Prázný, M. Kalousová, J. Kvasnička, L. Landová, T. Zima, J. Škrha

**Affiliations:** ^1^Institute of Medical Biochemistry and Laboratory Diagnostics, First Faculty of Medicine, Charles University in Prague, General University Hospital in Prague, 128 08 Prague 2, Czech Republic; ^2^3rd Department of Internal Medicine, First Faculty of Medicine, Charles University in Prague, General University Hospital in Prague, 128 08 Prague 2, Czech Republic; ^3^Department of Internal Medicine, Ayos District Hospital, Yaounde, Cameroon

## Abstract

The aim of this study was to compare skin autofluorescence caused by advanced glycation end-products (AGEs) with biochemical markers of endothelial dysfunction and soluble receptor for AGEs (sRAGE) in patients with diabetes. Skin autofluorescence (AF) assessed by AGE-Reader was evaluated with sRAGE and other biochemical parameters in 88 patients with diabetes (47 Type 1/T1DM/ and 41 Type 2/T2DM/) and 20 controls. Skin AF was significantly higher in T1DM and T2DM in comparison to controls (2.39 ± 0.54, 2.63 ± 0.73 versus 1.96 ± 0.33 AU; *P* < 0.0001). Positive correlation of AF with sRAGE was detected in T1DM and T2DM (*r* = 0.37, *P* < 0.02 and *r* = 0.60, *P* < 0.0001), but not in controls. Significantly higher AF values were found in patients with positive albuminuria as compared to those with normal albuminuria. Similarly, higher AF was detected in patients with endothelial dysfunction expressed by vWF, ICAM-1, and VCAM-1. Multiple regression analysis revealed independent association of skin AF with age, sRAGE, and albumin-creatinine ratio in patients with diabetes (*R* ^2^ = 0.38). Our study confirms that AF is elevated in patients with diabetes, especially with positive albuminuria and endothelial dysfunction. The strong and independent relationship between AF and sRAGE supports the idea that AF may reflect AGEs/RAGE interactions. The exact mechanism remains to be established.

## 1. Introduction

The accumulation of advanced glycation end-products (AGEs) belongs to the most important processes in the pathogenesis of long-term vascular complications in diabetes [1, 2]. AGEs are final products of nonenzymatic glycation of proteins, forming cross-links with collagen and other proteins, resulting in decreased vessel elasticity [3]. Cellular function and growth abilities are altered by AGEs as well [4]. In addition, AGEs activate receptors for advanced glycation end-products (RAGE) with subsequently accelerated inflammation [5, 6]. At the present time, a wide range of substances called alarmins, such as S100 calgranulins (e.g., EN-RAGE—also called S100A12), high mobility group box-1 (HMGB1), or amyloid-*β* peptide, is known to bind and activate RAGE more potently than AGEs alone [7]. Alarmins are present in physiological conditions, especially in acute inflammation, but their role is obviously also in promoting chronic inflammation as observed in diabetes, aging, atherosclerosis, tumorogenesis, or neurodegenerative disorders [8, 9]. On the contrary, the soluble form of RAGE (sRAGE) has been proposed as a protective biomarker elevated by RAGE activation [10–12].

The DCCT substudy showed that skin collagen glycation products are better predictors for diabetes complications than HbA_1c_ [13]. The need for skin biopsy was, however, a serious limitation in clinical monitoring. Other clinical studies have demonstrated that increased serum levels of AGEs are seen within 2–5 years of diabetes onset according to glycemic levels [14]. Assessment of serum AGEs was, however, rarely used in clinical practice, since expensive technics such as gas and liquid chromatography or mass spectrometry were necessary [15]. Another determination of fluorescent AGEs was based on spectrofluorometric detection [16], but this method was rather abandoned for nonspecificity. 

Recently, the AGE-Reader measuring skin autofluorescence was developed for noninvasive assessment of AGEs levels in the skin. A significant correlation between skin AGEs measured noninvasively and skin biopsy levels of collagen-linked fluorescence was found. Moreover, skin AF also correlated with serum levels of AGEs (pentosidine, CML, CEL) [17]. The results may reflect both metabolic and cardiovascular risks in patients with diabetes and thus could be routinely used in diabetes care [18, 19].

The aim of this study was to compare AGEs levels in the skin measured by AGE-Reader with biochemical markers of endothelial dysfunction (vWF, ICAM, VCAM, E-selectin, and P-selectin) and sRAGE levels in Type 1 and Type 2 patients with diabetes, to search for a relationship between AGE/RAGE axis and endothelial function.

## 2. Patients and Methods

### 2.1. Subjects

The whole group of 88 patients with diabetes consisted of 47 patients (27 males, 20 females) with Type 1 diabetes mellitus (T1DM) and 41 patients (25 males, 16 females) with Type 2 diabetes mellitus (T2DM), all from the diabetes outpatient clinic. Since the skin autofluorescence is age dependent [17], the subjects of roughly similar variation in calendar age were included. Their characteristics are shown in Table 1. All patients with severe hypertension, renal failure or liver impairment, neurodegenerative disorders, known malignancy, or infections, which could significantly influence laboratory variables, were excluded from the study. All patients with Type 1 diabetes were on intensified insulin treatment using 4 to 5 insulin injections, 20 patients with Type 2 diabetes were on metformin alone, 10 patients were on oral agents plus insulin, and the remaining 11 patients with Type 2 diabetes were treated by insulin only. Twenty T1DM and 30 T2DM were treated by statins. ACE inhibitors or AT1 blockers were used in 25 T1DM and 38 T2DM. Control group consisted of 20 healthy persons (5 males, 15 females) of comparable age. 

The study was performed in accordance with principles of the Declaration of Helsinki and was approved by local Ethics Committee of the General University Hospital and First Faculty of Medicine. All examined persons gave informed consent prior to being enrolled into the study.

### 2.2. Skin Autofluorescence

Skin AF was assessed on the ventral site of the forearm by AGE-Reader (DiagnOptics BV, Groningen, The Netherlands), as described previously [20]. Skin AF is calculated by dividing the mean value of the emitted light intensity per nm between 420 and 600 nm by the mean value of the excitation light intensity per nm between 300 and 420 nm, expressed in arbitrary units (AU). The intra-individual percent Altman error is 5.0% on a single day and 5.9% for seasonal changes [17]. 

### 2.3. Biochemical Methods

Fasting blood samples were collected between 7.00 and 8.00 AM from the cubital vein. Routine biochemical parameters were determined in fresh samples, whereas special biochemical analyses were done in serum frozen at −80°C until the assay measurement.

Serum concentrations of soluble RAGE were measured according to the manufacturer's protocol using sandwich ELISA (Quantikine, RD Systems, Minneapolis, MN, USA). In this assay, the plate is coated with a monoclonal antibody against RAGE while a polyclonal antibody is used for detection. This assay measures both C-truncated RAGE that has been cleaved from the cell surface, and esRAGE as well [21]. Endothelial dysfunction was evaluated by serum concentrations of specific markers, such as adhesion molecules (ICAM, VCAM, E-selectin, and P-selectin) and vWF, as proposed previously [22, 23]. Cell adhesion molecules Human sP-selectin/CD62P, Human sE-selectin/CD62E, Human sICAM-1/CD54, and Human sVCAM-1 were estimated by ELISA kits manufactured by RD System Europe (Abingdon, UK). von Willebrand factor (vWF) was determined by Corgenix (Broomfield, USA). 

Routine biochemical parameters including urea, creatinine, transaminases, alkaline phosphatase, *γ*-glutamyltransferase, total cholesterol, and triglycerides were determined by standard methods in central laboratory on Modular Roche analyzer. Fasting plasma glucose was determined by glucose oxidase method on glucose analyzer Super GLAmbulance (Dr. Müller Gerätebau, Freital, Germany); glycated hemoglobin HbA_1c_ was measured by HPLC on Variant II (Biorad, France) and expressed according to IFCC (normal values are 28–40 mmol/mol). 

Albuminuria was determined after exclusion of urinary infection in the second morning sample by radioimmunoassay using commercial kits (Immunotech, Czech Republic) and albumin/creatinine ratio (ACR) was calculated. Positive albuminuria (A+) was recognized when albumin/creatinine ratio >3 g/mol creatinine was found. Logarithmically transformed data were used for further analysis because log-normal distribution of the values was found. Renal functions were evaluated by estimated glomerular filtration rate (eGFR) calculated by MDRD formula [24].

### 2.4. Statistical Analysis

Results of biochemical parameters are expressed as mean ± standard deviation (SD) or as median (interquartile range). Differences between the groups were analyzed by one-way analysis of variance (ANOVA) followed by post hoc test analysis. Correlations between variables were assessed by Pearson's or Spearman's coefficient as appropriate. Variables correlated with AF at *P* < 0.05 were included in the multivariate analysis. Multiple linear regression analysis was carried out to disclose the statistically independent associations of AF with other variables. For statistical analyses, the software “Statistica 10” by StatSoft Inc. was used. The results were considered as statistically significant at *P* < 0.05.

## 3. Results

The characteristics of patients with Type 1 and Type 2 diabetes and control persons are shown in Table 1. The influence of gender as an independent variable on measured variables was not detected. Diabetes control was not significantly different between Type 1 and Type 2 diabetic patients (HbA_1c_  74 ± 14 versus 71 ± 21 mmol/mol, NS). Significantly higher fructosamine was observed in both T1DM and T2DM as compared to controls (369 ± 65 and 305 ± 47 versus 237 ± 20 *μ*mol/L, *P* < 0.0001). 

The results of skin AF and special biochemical variables in T1DM and T2DM as well as in control group are shown in Table 2. Skin AF was significantly higher in patients with T1DM and T2DM in comparison to controls (2.39 ± 0.54, 2.63 ± 0.73 versus 1.96 ± 0.33 AU; *P* < 0.0001), but the difference between T1DM and T2DM was not significant (Table 2). No significant differences in sRAGE concentration and in other special biochemical variables were found between both types of diabetes. 

Type 1 and Type 2 diabetic patients either with or without microalbuminuria were evaluated together (Table 3). Skin AF was higher in diabetic patients with microalbuminuria (A+) compared to those with normoalbuminuria (A−). Similarly, elevated sRAGE concentrations were found in microalbuminuric as compared to normoalbuminuric patients. 

Regarding endothelial dysfunction (ED), vWF, ICAM-1, and VCAM-1 were selected as indicators. All patients with diabetes were divided according to the activity of vWF into ED_vWF_^−^ (vWF < 110%) and ED_vWF_^+^ (vWF > 110%) subgroups, according to the concentration of ICAM-1 into ED_ICAM-1_^−^ (ICAM-1 < 300 *μ*g/L) and ED_ICAM-1_^+^ (ICAM-1 > 300 *μ*g/L), and according to the concentration of VCAM-1 into ED_VCAM-1_^−^ (VCAM-1 < 800 *μ*g/L) and ED_VCAM-1_^+^ (VCAM-1 > 800 *μ*g/L), respectively. Significant differences were observed in the skin AF both between ED_vWF_^−^ and ED_vWF_^+^ subgroups (2.27 ± 0.52 versus 2.63 ± 0.55 AU, *P* < 0.02), between ED_ICAM-1_^−^ and ED_ICAM-1_^+^ subgroups (2.31 ± 0.50 versus 2.91 ± 0.48 AU, *P* < 0.001), and between ED_VCAM-1_^−^ and ED_VCAM-1_^+^ subgroups (2.30 ± 0.42 versus 2.61 ± 0.63 AU, *P* < 0.05). On the contrary, differences in HbA_1c_ in ED_vWF_^−^ versus ED_vWF_^+^ (73 ± 17 versus 80 ± 19 mmol/mol, NS), ED_ICAM-1_^−^ versus ED_ICAM-1_^+^ (74 ± 17 versus 83 ± 20 mmol/mol, NS), and ED_VCAM-1_^−^ versus ED_VCAM-1_^+^ (74 ± 18 versus 79 ± 17 mmol/mol, NS) were not significant.

Differences in the skin AF observed in patients using antihypertensive medication (ACE inhibitors or AT1 blockers) or statins did not reach statistical significance. Similarly, the effect of antihyperglycemic therapy on AF levels was evaluated, but there were no significant differences in the skin AF between patients on metformin alone, metformin + insulin, or insulin alone.

**Figure 1:**
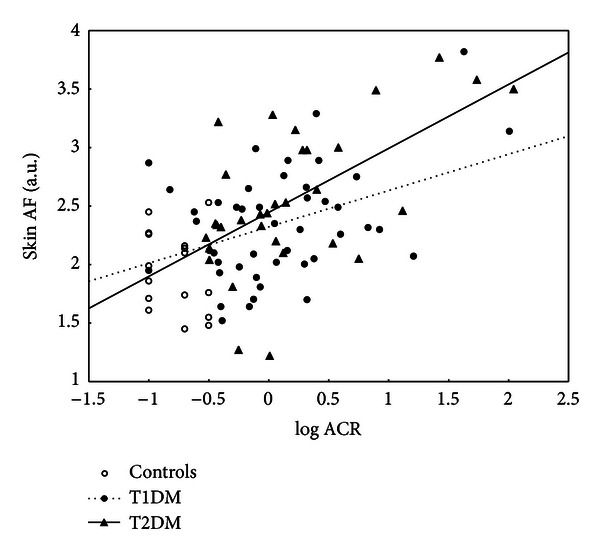
Relationship of skin AF and albuminuria expressed by ACR in controls, T1DM, and T2DM.

Univariate correlation analysis showed several relationships between skin AF and biochemical variables, and significant correlations are shown in Table 4. Firstly, skin AF was related to diabetes control expressed by HbA_1c_ in T2DM (*r* = 0.41, *P* < 0.01), but not in T1DM (*r* = 0.22, NS). Neither relationship of AF to fructosamine nor to fasting plasma glucose concentrations were observed in any group. Skin AF was age dependent both in controls (*r* = 0.73, *P* < 0.0005) and in T1DM (*r* = 0.54, *P* < 0.0001), but not significantly in T2DM (*r* = 0.22, NS). In T1DM and T2DM patients skin AF correlated with albuminuria expressed as albumin/creatinine ratio (*r* = 0.34, *P* < 0.05; *r* = 0.44, *P* < 0.01) (Figure 1) or inversely with eGFR (*r* = −0.48, *P* < 0.001; *r* = −0.30, *P* = 0.05).

**Figure 2:**
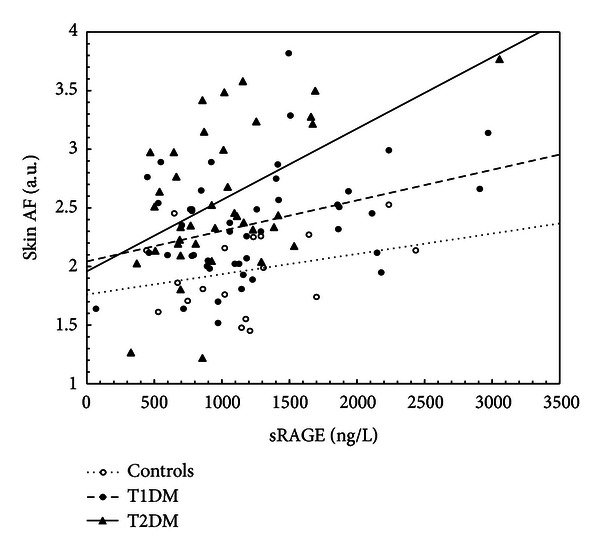
Relationship of skin AF and sRAGE in controls, T1DM and T2DM.

In T1DM but not T2DM, positive relationship was found between skin AF and ICAM-1 (*r* = 0.61, *P* < 0.001) and vWF (*r* = 0.52, *P* < 0.005). Interestingly, a significant positive correlation of skin AF with sRAGE was detected in T1DM and even stronger in T2DM (*r* = 0.37, *P* < 0.02 and *r* = 0.60, *P* < 0.0001), but not in controls (*r* = 0.29, NS) (Figure 2). If the patients with diabetes were divided into subgroups without (A−) and with (A+) microalbuminuria according to ACR, an important positive correlation of skin AF with sRAGE was observed in A+ subgroup (*r* = 0.61, *P* < 0.006), while it was less expressed in A− subgroup (*r* = 0.27, *P* < 0.03). In both patients with diabetes and controls, no relationship of autofluorescence with sex, blood pressure, or BMI was observed.

Multiple linear regression analysis was performed to establish independent associations of skin AF with those variables in which skin AF was correlated in univariate analysis at *P* < 0.05. Skin AF was independently associated with age (*β* = 0.33; *P* < 0.002), sRAGE (*β* = 0.36; *P* < 0.0002), and albuminuria (*β* = 0.23; *P* < 0.02) when the patients with both types of diabetes were analyzed together (*R* ^2^ = 0.38). In T1DM and T2DM evaluated separately skin AF was associated independently with age (*β* = 0.38; *P* < 0.01) in T1DM (*R* ^2^ = 0.40) and with sRAGE (*β* = 0.53; *P* < 0.004) in T2DM (*R* ^2^ = 0.44). In controls, skin AF was independently associated only with age (*β* = 0.55; *P* < 0.03; *R* ^2^ = 0.62). Other parameters such as gender, blood pressure, BMI, total cholesterol, HbA_1c_, or fructosamine were not associated with skin AF in our multiple linear regression analysis.

## 4. Discussion

This is the first study describing significant correlation of skin autofluorescence with sRAGE levels in both T1DM and T2DM. This relationship significantly depends on the severity of diabetic nephropathy. Previously, elevated levels of skin AGEs and sRAGE were observed in patient with systemic lupus erythematosus [25], and a significant correlation was described between skin AF and sRAGE in one Japanese with stable coronary artery disease [26]. However, the strong relationship of skin AF and sRAGE in patients with diabetes has not been reported yet. The AGEs/RAGE interaction results in the generation of intracellular oxidative stress, an enhanced inflammatory response and upregulated RAGE-ligand interaction [7]. On the contrary, sRAGE has been reported to act as a decoy for RAGE ligands. But this protective role has been recently supposed to be very limited, since the concentration of sRAGE is many times lower than the concentration of RAGE ligands [27]. In our study, the total pool of soluble RAGE with Quantikine immunoassay was measured and therefore we cannot discern whether the different variants of sRAGE (esRAGE or C-truncated RAGE) have some specific association to the skin autofluorescence. However, strong correlation of sRAGE and esRAGE was reported previously (*r* = 0.95, *P* < 0.001) [28].

Our results confirmed higher skin autofluorescence in T1DM and T2DM than in controls. Similar results were reported previously demonstrating significant relationship between skin autofluorescence and fluorescent and nonfluorescent skin AGEs content [17]. The increased skin accumulation of AGEs in patients with diabetes in consequence of chronic hyperglycemia and/or oxidative stress has been proposed. In our study we did not perform skin biopsies to verify the relationship between skin autofluorescence and tissue AGEs content. Moreover, it should be pointed out that AGEs accumulation in tissues and skin AF, respectively, need not to reflect AGEs levels in circulation, as observed by Nienhuis et al. [25]. Patients in our study with developed microalbuminuria as an early clinical sign of nephropathy had significantly higher skin autofluorescence as compared to those without renal changes also after adjustment to renal functions. The same was shown in the follow-up study by Gerrits et al. [29]. 

In the present study we found a positive association between skin AF levels and HbA_1c_ in T2DM, but not in T1DM. Such association was not found in T2DM in a cohort by Gerrits et al. [30]. Since the relatively short turnover time of hemoglobin [31] as compared to skin AGEs formation and degradation, the HbA_1c_ values cannot always reflect AGEs accumulation. In addition, neither shortly glycated proteins expressed by fructosamine nor fasting plasma glucose were related to AF in our study. 

Endothelial dysfunction indicated by elevation of vWF, ICAM-1, or VCAM-1 was associated with significantly higher AF than in diabetic patients with normal levels of these parameters. This is the first observation to our knowledge when AF is higher in diabetic patients with endothelial dysfunction. Elevated vWF and VCAM-1 concentrations as markers of endothelial activation were found together with higher skin autofluorescence in patients with rheumatoid arthritis [32].

There are some limitations of this study. Firstly, we have not measured serum levels of AGEs, since they were detected by AGE-Reader. Some nonfluorescent AGEs are not detected by AGE-Reader, but it has been reported previously that skin autofluorescence strongly correlated with skin levels of nonfluorescent AGE [17]. Moreover, the total specificity and sensitivity of this tool were already reported [17]. Secondly, all subjects in this study were Caucasians and therefore the results cannot be applied in general, since ethnic determinants may influence AF and other parameters. In addition, the total number of patients in each group was limited. Finally, multiple factors such as age, ethnic determinants, *AGER* gene polymorphisms, or renal functions may influence the sRAGE levels [11, 33, 34]. Similarly, since skin AF is age dependent [17, 35]; we involved younger T2DM predominantly to maintain age-matched groups for further analysis. Despite the fact that sRAGE levels are modified in the presence of microalbuminuria or slightly decreased renal functions, patients with such complications were not excluded from this study although severe renal impairment was not present in any of our patients. 

In conclusion, skin AF is an applicable variable for AGEs accumulation assessment. Our study confirms that skin autofluorescence is elevated in patients with diabetes and the most significant increase has been found in patients with microangiopathy expressed mainly by albuminuria. Similar results were found in sRAGE concentrations. The strong relationship between AF and sRAGE supports the idea of mutual interactions between RAGE, AGEs, and other RAGE ligands. Endothelial dysfunction could be an important factor accelerating the AGEs accumulation as well. The exact mechanism of such interactions in patients with diabetes remains to be elucidated. 

## Figures and Tables

**Table 1:** Characteristics of patients with Type 1 (T1DM), Type 2 (T2DM), and controls.

	T1DM	T2DM	Controls	ANOVA
	(*n* = 47)	(*n* = 41)	(*n* = 20)	*P* value
Age (yrs)	46 (25–76)	58 (39–77)	46 (24–65)	
Sex (% of males)	57	61	25	
Duration of DM (yrs)	14 (3–52)	9 (2–31)	—	
BMI (kg/m^2^)	25.9 ± 2.9^z^	33.1 ± 10.0^c^	25.5 ± 3.4	**<0.0001**
SBP (mm Hg)	131 ± 10^x^	138 ± 13^a^	129 ± 12	**<0.01**
DBP (mm Hg)	78 ± 7	79 ± 8	75 ± 6	0.24
Cholesterol (mmol/L)	4.69 ± 0.79	4.54 ± 0.92	4.48 ± 0.61	0.53
Triglycerides (mmol/L)	1.04 ± 0.53^z^	1.92 ± 0.92^c^	1.09 ± 0.58	**<0.0001**
Glucose (mmol/L)	7.9 ± 4.0^c^	9.3 ± 3.5^c^	4.5 ± 0.6	**<0.0001**
HbA_1c_ (mmol/mol, IFCC)	74 ± 14^c^	71 ± 21^c^	39 ± 5	**<0.0001**
Fructosamine (*μ*mol/L)	369 ± 65^cz ^	305 ± 47^c^	237 ± 20	**<0.0001**
CRP (mg/L)	3.0 ± 1.9^a^	4.1 ± 2.1^a^	1.4 ± 1.8	**<0.005**
Albumin/creatinine (g/mol)	1.17^a^ (0.29–4.79)	1.86^c^ (0.36–9.55)	0.45 (0.19–1.14)	**<0.01**
Creatinine (*μ*mol/L)	85.8 ± 33.4	88.9 ± 22.2^a^	72.3 ± 12.8	0.10
eGFR (ml·s^−1^·(1.73 m^2^)^−1^)	1.3 ± 0.3	1.2 ± 0.3	1.4 ± 0.3	0.05

Results are means ± SD, or means with 1 SD range, in age and duration of DM medians with ranges. One-way ANOVA was performed, with *P* values in the last column of the table. Statistical significance expressed by LSD post hoc test between DM and control persons: ^a^ *P* < 0.05, ^b^ *P* < 0.01, and ^c^ *P* < 0.001, and between T1DM and T2DM: ^x^ *P* < 0.05, ^y^ *P* < 0.01, and ^z^ *P* < 0.001.

Abbreviations: DM: diabetes mellitus; BMI: body mass index; SBP: systolic blood pressure; DBP: diastolic blood pressure; CRP: C-reactive peptide; eGFR: estimated glomerular filtration rate.

**Table 2:** Skin autofluorescence and novel biochemical parameters.

	T1DM	T2DM	Controls	ANOVA
	(*n* = 47)	(*n* = 41)	(*n* = 20)	*P* value
AF (AU)	2.39 ± 0.54^b^	2.63 ± 0.73^c^	1.96 ± 0.33	**<0.0001**
sRAGE (ng/L)	1250 ± 627	1040 ± 567	1163 ± 537	0.26
vWF (%)	114 ± 48	127 ± 56^a^	93 ± 25	**0.038**
sICAM-1 (*μ*g/L)	269 ± 89	266 ± 79	223 ± 82	0.10
sVCAM-1 (*μ*g/L)	907 ± 498^c^	816 ± 271^c^	392 ± 91	**<0.0001**
P-selectin (*μ*g/L)	113 ± 36^b^	101 ± 35^c^	145 ± 52	**0.0004**
E-selectin (*μ*g/L)	29 ± 16^a^	36 ± 16	41 ± 15	**0.011**

Results are means ± SD, or means with 1 SD range. One-way ANOVA was performed, with *P* values in the last column of the table. Statistical significance expressed by LSD post hoc test between DM and control persons: ^a^ *P* < 0.05, ^b^ *P* < 0.01, and ^c^ *P* < 0.001.

Abbreviations: AF: autofluorescence; AU: arbitrary unit; sRAGE: soluble receptor for advanced glycation end-products; vWF: von Willebrand factor; sICAM: intercellular adhesion molecule; sVCAM: vascular cell adhesion molecule.

**Table 3:** Skin autofluorescence and novel biochemical parameters in subgroups without (A−) and with (A+) (micro)albuminuria according to ACR.

	A+	A−	Controls	ANOVA
	(*n* = 19)	(*n* = 69)	(*n* = 20)	*P* value
AF (AU)	2.84 ± 0.80^cx^	2.41 ± 0.56^b^	1.96 ± 0.33	**<0.0001**
Albumin/creatinine (g/mol)	13.2^cz^ (3.9–44.0)	0.7 (0.3–1.6)	0.5 (0.2–1.1)	**<0.0001**
HbA_1c_ (mmol/mol, IFCC)	90 ± 18^cz^	68 ± 14^c^	39 ± 5	**<0.0001**
sRAGE (ng/L)	1457 ± 754^x^	1066 ± 532	1163 ± 537	**<0.05**
vWF (%)	137 ± 47^b^	114 ± 45	93 ± 25	**0.007**
sICAM (*μ*g/L)	324 ± 102^cy^	251 ± 70	223 ± 82	**0.0003**
sVCAM (*μ*g/L)	965 ± 408^c^	832 ± 400^c^	392 ± 91	**<0.0001**
P-selectin (*μ*g/L)	102 ± 33^b^	109 ± 37^b^	145 ± 52	**0.0008**
E-selectin (*μ*g/L)	39 ± 18	31 ± 15^a^	41 ± 15	**0.016**

Results are means ± SD, or means with 1 SD range. One-way ANOVA was performed, with *P* values in the last column of the table. Statistical significance expressed by LSD post hoc test between DM and control persons: ^a^ *P* < 0.05, ^b^ *P* < 0.01, and ^c^ *P* < 0.001 and between A+ and A−: ^x^ *P* < 0.05, ^y^ *P* < 0.01, ^z^ *P* < 0.001.

Abbreviations**: **AF: autofluorescence; AU: arbitrary unit; HbA_1c_: glycated hemoglobin; sRAGE: soluble receptor for advanced glycation end-products; vWF: von Willebrand factor; sICAM: intercellular adhesion molecule; sVCAM: vascular cell adhesion molecule.

**Table 4:** The univariate significant correlations between skin autofluorescence and biochemical parameters.

	Skin AF		
	T1DM	T2DM	Controls
Duration of DM	0.25	**0.38**	—
	(NS)	(0.01)	
Age	**0.53**	0.22	**0.73**
	(0.0001)	(NS)	(0.0004)
HbA_1c_	0.22	**0.41**	0.33
	(NS)	(0.007)	(NS)
sRAGE	**0.37**	**0.60**	0.29
	(0.02)	(0.00003)	(NS)
sICAM	**0.61**	0.16	0.37
	(0.0007)	(NS)	(NS)
vWF	**0.52**	0.28	**0.63**
	(0.005)	(NS)	(0.004)
Creatinine	**0.34**	0.25	−0.28
	(0.02)	(NS)	(NS)
ACR	**0.34**	**0.44**	0.22
	(0.02)	(0.007)	(NS)
eGFR	**−0.48**	**−0.30**	−0.33
	(0.001)	(0.05)	(NS)

*r* in the first line of the cell, *P* in the brackets.

Abbreviations: AF: autofluorescence; AU: arbitrary unit; HbA_1c_: glycated hemoglobin; sRAGE: soluble receptor for advanced glycation end-products; vWF: von Willebrand factor; sICAM: intercellular adhesion molecule; ACR: albumin-creatinine ratio; eGFR: estimated glomerular filtration rate.

## Abbreviations

AGEs: Advanced glycation end-products
AF: Autofluorescence
RAGE: Receptor for advanced glycation end-products
HbA_1c_: Glycated hemoglobin
vWF: Von Willebrand factor
ICAM: Intercellular adhesion molecule
VCAM: Vascular cell adhesion molecule
ACR: Albumin-creatinine ratio
eGFR: Estimated glomerular filtration rate
BMI: Body mass index
SBP: Systolic blood pressure
DBP: Diastolic blood pressure
CML: Carboxymethyllysine
CEL: Carboxyethyllysine.

## References

[1] Genuth S, Sun W, Cleary P (2005). Glycation and carboxymethyllysine levels in skin collagen predict the risk of future 10-year progression of diabetic retinopathy and nephropathy in the diabetes control and complications trial and epidemiology of diabetes interventions and complications participants with type 1 diabetes. *Diabetes*.

[2] Brownlee M (2005). The pathobiology of diabetic complications: a unifying mechanism. *Diabetes*.

[3] Zieman SJ, Melenovsky V, Clattenburg L (2007). Advanced glycation endproduct crosslink breaker (alagebrium) improves endothelial function in patients with isolated systolic hypertension. *Journal of Hypertension*.

[4] Giardino I, Edelstein D, Brownlee M (1994). Nonenzymatic glycosylation in vitro and in bovine endothelial cells alters basic fibroblast growth factor activity. A model for intracellular glycosylation in diabetes. *Journal of Clinical Investigation*.

[5] Hofmann MA, Drury S, Fu C (1999). RAGE mediates a novel proinflammatory axis: a central cell surface receptor for S100/calgranulin polypeptides. *Cell*.

[6] Haslbeck KM, Schleicher E, Bierhaus A (2005). The AGE/RACE/NF-*κ*B pathway may contribute to the pathogenesis of polyneuropathy in impaired glucose tolerance (IGT). *Experimental and Clinical Endocrinology and Diabetes*.

[7] Yao D, Brownlee M (2010). Hyperglycemia-induced reactive oxygen species increase expression of the receptor for advanced glycation end products (RAGE) and RAGE ligands. *Diabetes*.

[8] Bierhaus A, Nawroth PP (2009). Multiple levels of regulation determine the role of the receptor for AGE (RAGE) as common soil in inflammation, immune responses and diabetes mellitus and its complications. *Diabetologia*.

[9] Yan SF, Yan SD, Ramasamy R, Schmidt AM (2009). Tempering the wrath of RAGE: an emerging therapeutic strategy against diabetic complications, neurodegeneration, and inflammation. *Annals of Medicine*.

[10] Katakami N, Matsuhisa M, Kaneto H (2008). Endogenous secretory RAGE but not soluble RAGE is associated with carotid atherosclerosis in type 1 diabetes patients. *Diabetes and Vascular Disease Research*.

[11] Yan SF, Ramasamy R, Schmidt AM (2010). Soluble RAGE: therapy and biomarker in unraveling the RAGE axis in chronic disease and aging. *Biochemical Pharmacology*.

[12] Vazzana N, Santilli F, Cuccurullo C, Davì G (2009). Soluble forms of RAGE in internal medicine. *Internal and Emergency Medicine*.

[13] Monnier VM, Bautista O, Kenny D (1999). Skin collagen glycation, glycoxidation, and crosslinking are lower in subjects with long-term intensive versus conventional therapy of type 1 diabetes: relevance of glycated collagen products versus HbA(1c) as markers of diabetic complications. *Diabetes*.

[14] Chiarelli F, De Martino M, Mezzetti A (1999). Advanced glycation end products in children and adolescents with diabetes: relation to glycemic control and early microvascular complications. *Journal of Pediatrics*.

[15] Meerwaldt R, Lutgers HL, Links TP (2007). Skin autofluorescence is a strong predictor of cardiac mortality in diabetes. *Diabetes Care*.

[16] Münch G, Keis R, Weßels A (1997). Determination of advanced glycation end products in serum by fluorescence spectroscopy and competitive ELISA1. *European Journal of Clinical Chemistry and Clinical Biochemistry*.

[17] Meerwaldt R, Graaf R, Oomen PHN (2004). Simple non-invasive assessment of advanced glycation endproduct accumulation. *Diabetologia*.

[18] Sattar N (2012). Biomarkers for diabetes prediction, pathogenesis or pharmacotherapy guidance? Past, present and future possibilities. *Diabetic Medicine*.

[19] Monami M, Lamanna C, Gori F, Bartalucci F, Marchionni N, Mannucci E (2008). Skin autofluorescence in type 2 diabetes: beyond blood glucose. *Diabetes Research and Clinical Practice*.

[20] Mulder DJ, Van De Water T, Lutgers HL (2006). Skin autofluorescence, a novel marker for glycemic and oxidative stress-derived advanced glycation end-products: an overview of current clinical studies, evidence, and limitations. *Diabetes Technology and Therapeutics*.

[21] Yonekura H, Yamamoto Y, Sakurai S (2003). Novel splice variants of the receptor for advanced glycation end-products expressed in human vascular endothelial cells and pericytes, and their putative roles in diabetes-induced vascular injury. *Biochemical Journal*.

[22] Haim M, Tanne D, Boyko V (2002). Soluble intercellular adhesion molecule-1 and long-term risk of acute coronary events in patients with chronic coronary heart disease: data From the Bezafibrate Infarction Prevention (BIP) Study. *Journal of the American College of Cardiology*.

[23] Widlansky ME, Gokce N, Keaney JF, Vita JA (2003). The clinical implications of endothelial dysfunction. *Journal of the American College of Cardiology*.

[24] Levey AS, Bosch JP, Lewis JB, Greene T, Rogers N, Roth D (1999). A more accurate method to estimate glomerular filtration rate from serum creatinine: a new prediction equation. *Annals of Internal Medicine*.

[25] Nienhuis HL, De leeuw K, Bijzet J (2008). Skin autofluorescence is increased in systemic lupus erythematosus but is not reflected by elevated plasma levels of advanced glycation end-products. *Rheumatology*.

[26] Mulder DJ, van Haelst PL, Gross S (2008). Skin autofluorescence is elevated in patients with stable coronary artery disease and is associated with serum levels of neopterin and the soluble receptor for advanced glycation end products. *Atherosclerosis*.

[27] Humpert PM, Djuric Z, Kopf S (2007). Soluble RAGE but not endogenous secretory RAGE is associated with albuminuria in patients with type 2 diabetes. *Cardiovascular Diabetology*.

[28] Kalousová M, Jáchymová M, Oto M (2007). Receptor for advanced glycation end products—soluble form and gene polymorphisms in chronic haemodialysis patients. *Nephrology Dialysis Transplantation*.

[29] Gerrits EG, Lutgers HL, Kleefstra N (2008). Skin autofluorescence: a tool to identify type 2 diabetic patients at risk for developing microvascular complications. *Diabetes Care*.

[30] Gerrits EG, Lutgers HL, Kleefstra N, Groenier KH, Smit AJ, Gans RO (2008). Skin advanced glycation end product accumulation is poorly reflected by glycemic control in type 2 diabetic patients (ZODIAC-9). *Journal of Diabetes Science and Technology*.

[31] Furth AJ (1997). Glycated proteins in diabetes. *British Journal of Biomedical Science*.

[32] de Groot L, Hinkema H, Westra J, Smit AJ, Kallenberg CGM, Bijl M (2011). Advanced glycation end-products are increased in rheumatoid arthritis patients with controlled disease. *Arthritis Research & Therapy*.

[33] Kalousova M, Hodkova M, Kazderova M, Fialova J, Tesar V, Dusilova-Sulkova S (2006). Soluble receptor for advanced glycation end products in patients with decreased renal function. *American Journal of Kidney Diseases*.

[34] Kankova K, Kalousova M, Hertlova M, Krusova D, Olsovsky J, Zima T (2008). Soluble RAGE, diabetic nephropathy and genetic variability in the AGER gene. *Archives of Physiology and Biochemistry*.

[35] Lutgers HL, Gerrits EG, Graaff R (2009). Skin autofluorescence provides additional information to the UK Prospective Diabetes Study (UKPDS) risk score for the estimation of cardiovascular prognosis in type 2 diabetes mellitus. *Diabetologia*.

